# Novel patient risk factors and validation of a difficulty scoring system in laparoscopic repeat hepatectomy

**DOI:** 10.1038/s41598-019-54099-w

**Published:** 2019-11-27

**Authors:** Yukiyasu Okamura, Yusuke Yamamoto, Teiichi Sugiura, Takaaki Ito, Ryo Ashida, Katsuhisa Ohgi, Katsuhiko Uesaka

**Affiliations:** Division of Hepato-Biliary-Pancreatic Surgery, Shizuoka Cancer Center Hospital, Shizuoka, Japan

**Keywords:** Liver, Liver cancer

## Abstract

The indications for laparoscopic liver resection have expanded; however, the safety and benefits of laparoscopic repeat hepatectomy (LRH) remain unclear. We retrospectively reviewed data from 137 patients who underwent partial hepatectomy or left lateral sectionectomy without thoracotomy. We compared patients’ clinical factors using a difficulty scoring system for LRH. We defined factors associated with blood loss volumes in the 75th percentile or above as risk factors for bleeding in open repeat hepatectomy, and determined whether these factors were useful for LRH risk assessment. Open repeat hepatectomy and LRH was performed in 96 and 41 patients, respectively. Four of 41 (9.8%) patients undergoing LRH were converted to laparotomy. Blood loss volume was significantly greater in the intermediate-risk group than in the low-risk group (*P* = 0.046). Multivariate analysis revealed that the presence of tumours located adjacent and caudal or dorsal to the primary tumour site was an independent risk factor for bleeding in LRH (odds ratio 3.21, 95% confidence interval 1.16–8.88, *P* = 0.024). Our study validated the usefulness of a difficulty scoring system, identified patient factors that predicted the difficulty of LRH, and presented a novel difficulty scoring system for LRH based on an existing difficulty scoring system.

## Introduction

Repeat hepatectomy is an effective treatment for liver tumours, including hepatocellular carcinoma (HCC)^1,2^ and colorectal metastatic tumours^3,4^. However, repeat hepatectomy is generally more technically difficult than primary hepatectomy because of adhesions and anatomical changes caused by the previous resection.

Laparoscopic techniques and instruments have progressed over the last two decades, and laparoscopic liver resection (LLR) is now frequently performed to treat liver malignant tumours worldwide. The National Clinical Database in Japan demonstrated that LLR is associated with less blood loss, fewer postoperative complications, and a shorter duration of postoperative hospitalization than conventional open hepatectomy^5^. As a result, the indications for LLR have been expanded, and laparoscopic repeat hepatectomy (LRH) has been introduced in several institutions. However, the safety and outcomes of LRH have not been fully assessed, as only a small number of studies with small sample sizes have been published^6–10^.

Ban *et al*.^11^ introduced a difficulty scoring system to assess the difficulty of LLR, and some studies have validated this scoring system^12–15^. However, it is difficult to use the scoring system to evaluate the difficulty of LRH, as the system does not assess factors related to repeat hepatectomy.

To identify the factors affecting intraoperative blood loss in LRH, we retrospectively analysed data from patients undergoing open repeat hepatectomy and evaluated whether the patient factors indicative of a high risk of blood loss in open repeat hepatectomy were useful for risk assessment in LRH. We also validated the utility of the difficulty scoring system proposed by Ban *et al*.^11^, modified the system to include our novel patient-related risk factors, and then evaluated the modified system for assessing the risk of bleeding in patients undergoing LRH.

## Results

### Patient characteristics

Among the 137 patients who underwent partial hepatectomy or left lateral sectionectomy without thoracotomy, 41 patients underwent LRH, while 96 underwent open repeat hepatectomy (Table 1). Four patients undergoing LRH (9.8%) were converted to laparotomy because the target tumour could not be detected under laparoscopic surgery (N = 2), severe adhesions were present (N = 1), or haemorrhage occurred (N = 1). Repeat hepatectomy was performed in 46 patients with HCC and 91 patients with metastatic tumours. HCC was significantly more frequently treated via LRH than via open repeat hepatectomy (46.3% vs. 28.1%, respectively; *P* = 0.049). Compared with the patients who underwent open repeat hepatectomy, the patients who underwent LRH had a significantly lower incidence of tumours located in segments 1, 7, or 8 (31.7% vs. 53.1%, respectively; *P* = 0.025) and a significantly lower incidence of tumours located ipsilateral to the previous hepatectomy site (46.3% vs. 78.9%, respectively; *P* = 0.001). Among the 41 patients who underwent LRH, 37 (90.2%) underwent surgery later in the study period (2015–2018).

**Table 1:** Comparison of the clinicopathological characteristics of the laparoscopic repeat hepatectomy group versus the open repeat hepatectomy group

	LRH	Open-Re-Hx	*P*
	n = 41	n = 96	
Age (years)^#^	65 (25–84)	67 (37–84)	0.851
Gender (male/female)	27/14	66/30	0.842
ASA-PS (3)	4 (9.8)	7 (7.3)	1.000
Tumour type (HCC/metastatic tumour)	19/22	27/69	0.049
Type of approach to the previous hepatectomy (Lap-Hx)	15 (36.6)	4 (4.2)	<0.001
Number of the previous hepatectomy (3 or more)	6 (14.6)	31 (32.3)	0.037
Pringle manoeuvre (performed)	17 (41.5)	52 (54.2)	0.195
Time of Pringle manoeuvre (minutes)^#^	0 (0–133)	16 (0–216)	0.374
Year of surgery (2015–2018)	37 (90.2)	41 (42.7)	<0.001
Tumour diameter (mm)^#^	15.5 (4.0–40)	20.0 (8.0–53)	0.035
Tumour number (multiple)	5 (12.2)	24 (25.0)	0.113
Tumour location (S1, S7 or S8)	13 (31.7)	51 (53.1)	0.025
Tumours located in the ipsilateral of the previous surgical site	19 (46.3)	75 (78.9)	0.001
Tumours located in the adjacent area on the cranial or dorsal side of the previous surgical site	14 (34.1)	47 (49.0)	0.134
Intraoperative blood loss (mL)^#^	60 (small amount-3,260)	311 (small amount-2,565)	<0.001
Operation time (minutes)^#^	178 (72–368)	203 (64–767)	0.099
Morbidity ≥ Clavien-Dindo grade 3a	1 (2.4)	6 (6.3)	0.674
R0 resection	36 of 39 (92.3)	91 of 93 (97.8)	0.153
Surgical margin (mm)^#^	4.5 (0–25)	3 (0–30)	0.066
Postoperative hospital stay (days)^#^	5 (4–41)	9 (4–35)	<0.001
Cirrhosis (present)	4 (9.8)	7 (7.3)	0.733

LRH, laparoscopic repeat hepatectomy; Open-Re-Hx, open repeat hepatectomy; ASA-PS, American Society of Anesthesiologists physical status; HCC, hepatocellular carcinoma; Lap-Hx, laparoscopic hepatectomy; S, segment. Values in parentheses are percentages unless indicated otherwise^#^.value is expressed as the median (range).

Compared with patients who underwent open repeat hepatectomy, patients who underwent LRH had a significantly smaller median volume of intraoperative blood loss (60 mL vs. 311 mL, *P* < 0.001) and a significantly shorter median postoperative hospital stay (5 days vs. 9 days, *P* < 0.001).

### Assessment of the usefulness of the Iwate difficulty scoring system in LRH

The Iwate difficulty scoring system was used to classify 15 and 25 patients who underwent LRH into the intermediate- and low-risk groups, respectively, (Table 2)^11^. A difficulty index value was not obtained in one patient with a tumour located in segment 1. There was no high-risk group with a score ≥7 because major hepatectomy is not performed via laparoscopic surgery in our institution.

**Table 2:** Comparisons of clinical factors of the patients who underwent LRH who were classified as having an intermediate or low risk of massive intraoperative blood loss in accordance with the Iwate difficulty scoring system.

	Intermediate (Difficulty index 4 or more)	Low (Difficulty index less than 4)	*P*
	n = 15	n = 25	
Age (years)^#^	68 (46–84)	65 (25–83)	0.761
Gender (male/female)	11/4	15/10	0.502
ASA-PS (3)	1 (6.7)	3 (12.0)	1.000
Tumour type (HCC/metastatic tumour)	7/8	12/13	1.000
Type of approach to the previous hepatectomy (Lap-Hx)	7 (46.7)	7 (28.0)	0.310
Number of the previous hepatectomy (3 or more)	4 (26.7)	2 (8.0)	0.174
Pringle manoeuvre (performed)	8 (53.3)	9 (36.0)	0.336
Time of Pringle manoeuvre (minutes)^#^	0 (0–133)	0 (0–108)	0.720
Year of surgery (2015–2018)	14 (93.3)	14 (56.0)	0.015
Tumour diameter (mm) ^#^	20 (7–38)	15 (4–40)	0.041
Tumour number (multiple)	3 (20.0)	2 (8.0)	0.345
Tumour location (S1, S7 or S8)	12 (80.0)	0	<0.001
Tumours located in the ipsilateral of the previous surgical site	12 (80.0)	7 (28.0)	0.003
Tumours located in the adjacent area on the cranial or dorsal side of the previous surgical site	11 (73.3)	3 (12.0)	<0.001
Intraoperative blood loss (mL)^#^	125 (small amount-3,260)	50 (small amount-784)	0.046
Operation time (minutes)^#^	218 (83–368)	174 (72–355)	0.083
Converted to laparotomy	3 (20.0)	1 (4.0)	0.139
Morbidity ≥ Clavien-Dindo grade 3a	1 (6.7)	0	0.375
Postoperative hospital stay (days)	6 (5–41)	5 (4–17)	0.016

LRH, laparoscopic repeat hepatectomy; ASA-PS, American Society of Anesthesiologists physical status; HCC, hepatocellular carcinoma; Lap-Hx, laparoscopic hepatectomy; S, segment.

Values in parentheses are percentages unless indicated otherwise^#^.value is expressed as the median (range).

Compared with the low-risk group, the intermediate-risk group had a significantly higher incidence of tumours located in segments 1, 7, or 8 (*P* < 0.001), significantly higher incidence of tumours ipsilateral to the previous surgical site (*P* = 0.003), and significantly higher incidence of tumours adjacent and cranial or dorsal to the previous surgical site (*P* < 0.001). The intermediate-risk group also had a significantly greater blood loss volume (*P* = 0.046) and longer duration of postoperative hospitalisation (*P = *0.016) than the low-risk group.

### Factors affecting massive blood loss in patients undergoing open repeat hepatectomy

**Figure 1:**
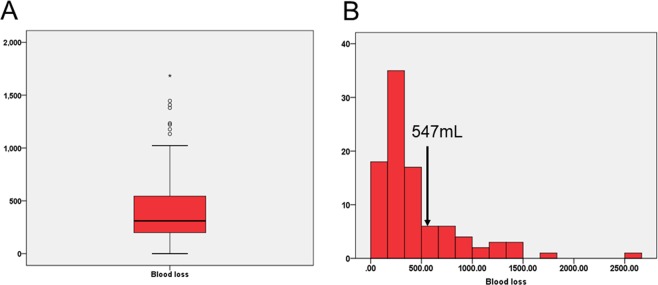
Bar graph showing intraoperative blood loss volume. (**A**) The ≥75th percentile for blood loss is indicated by the arrows (**B**).

The median intraoperative blood loss volume in the 96 patients who underwent open repeat hepatectomy was 311 mL (range: small immeasurable amount–2,565 mL), and the 75th percentile for intraoperative blood loss volume was 547 mL (Fig. 1). To identify the risk factors for massive intraoperative blood loss, we extracted factors associated with patients with a blood loss volume ≥the 75th percentile in open repeat hepatectomy. Multivariate analysis revealed that the presence of tumours located adjacent and cranial or dorsal to the previous surgical site (odds ratio: 3.21, 95% confidence interval: 1.16–8.88, *P* = 0.024) was an independent risk factor for massive intraoperative blood loss in open repeat hepatectomy (Table 3).

**Table 3:** Predictors of massive intraoperative blood loss in open repeat hepatectomy.

Variables	Univariate		Multivariate	
	Odds ratio (95% confidence interval)	*P*	Odds ratio (95% confidence interval)	*P*
**Massive blood loss**				
Age (>67 years)	0.54 (0.21–1.38)	0.198		
Gender (male)	1.50 (0.53–4.27)	0.447		
ASA-PS (3)	0.63 (0.07–5.70)	0.677		
Tumour type (metastatic tumour)	Not obtained	0.998		
Number of the previous hepatectomy (3 or more)	1.36 (0.52–3.59)	0.529		
Year of surgery (2015–2018)	1.40 (0.51–3.78)	0.513		
Tumour diameter (>20 mm)	0.94 (0.35–2.50)	0.901		
Tumour number (multiple)	2.96 (1.09–8.04)	0.032		
Tumour location (S1, S7 or S8)	1.32 (0.52–3.37)	0.556		
Tumours located in the ipsilateral of the previous surgical site (yes)	3.94 (0.85–18.4)	0.081		
Tumours located in the adjacent area on the cranial or dorsal side of the previous surgical site (yes)	3.40 (1.25–9.22)	0.016	3.21 (1.16–8.88)	0.024

ASA-PS, American Society of Anesthesiologists physical status; S, segment.

### Assessment of whether the risk factors for massive blood loss in open repeat hepatectomy are useful in predicting the difficulty of LRH

Among the 41 patients who underwent LRH, 14 patients (34.1%) had tumours located adjacent and cranial or dorsal to the previous surgical site. The difficulty index in patients with this risk factor was significantly higher than that in patients without this risk factor (median: 5 vs. 2.5, respectively; *P* = 0.001). The duration of the Pringle manoeuvre in patients with this risk factor was significantly longer than that in patients without this risk factor (median: 46 minutes vs. 0 minutes; *P* = 0.045). Patients with this risk factor had a significantly greater blood loss volume (median: 284 mL vs. 50 mL, *P* = 0.009) and longer operation time (median: 253 min. vs. 170 min, *P* = 0.002) than patients without this risk factor (Table 4). However, there was no significant difference between patients with and without this risk factor in the rate of conversion to laparotomy (*P* = 0.602).

**Table 4:** Comparisons of clinical factors of the patients who underwent LRH with and without tumours located in the adjacent area on the head or dorsal side of the primary site.

	Tumours located in the adjacent area on the cranial or dorsal side of the primary site	Tumours located in the adjacent area on the caudal or ventral side of the primary site	*P*
	n = 14	n = 27	
Age (years)^#^	72 (57–81)	65 (25–84)	0.154
Gender (male/female)	9/5	17/9	1.000
Difficulty index	5 (1–6)	2.5 (1–5)	0.001
ASA-PS (3)	0	4 (14.8)	0.278
Tumour type (HCC/metastatic tumour)	3/11	15/11	0.103
Type of approach to the previous hepatectomy (Lap-Hx)	6 (42.9)	8 (29.6)	0.501
Number of the previous hepatectomy (3 or more)	4 (28.6)	2 (7.4)	0.159
Pringle manoeuvre (performed)	9 (64.3)	8 (29.6)	0.102
Time of Pringle manoeuvre (minutes)^#^	46 (0–133)	0 (0–128)	0.045
Year of surgery (2015–2018)	14 (100)	14 (51.9)	0.003
Tumour diameter (mm)^#^	20 (4–40)	15 (6–30)	0.014
Tumour number (multiple)	4 (28.6)	5 (18.5)	0.043
Tumour location (S1, S7 or S8)	9 (64.3)	3 (11.1)	0.001
Tumours located in the ipsilateral of the primary site	13 (92.9)	6 (22.2)	<0.001
Intraoperative blood loss (mL) ^#^	284 (small amount-3,260)	50 (small amount-784)	0.009
Operation time (minutes)^#^	253 (129–368)	170 (72–355)	0.002
Converted to laparotomy	2 (14.3)	2 (7.4)	0.602
Morbidity ≥ Clavien-Dindo grade 3a	1 (7.1)	0	0.350
Postoperative hospital stay (days)	5 (4–41)	5 (4–17)	0.424

LRH, laparoscopic repeat hepatectomy; ASA-PS, The American Society of Anesthesiologists physical status; HCC, hepatocellular carcinoma; Lap-Hx, laparoscopic hepatectomy; S, segment.

Values in parentheses are percentages unless indicated otherwise; ^#^value is expressed as the median (range).

### Extent to which the presence of tumours located adjacent and cranial or dorsal to the previous surgical site affects the Iwate difficulty scoring system

**Figure 2:**
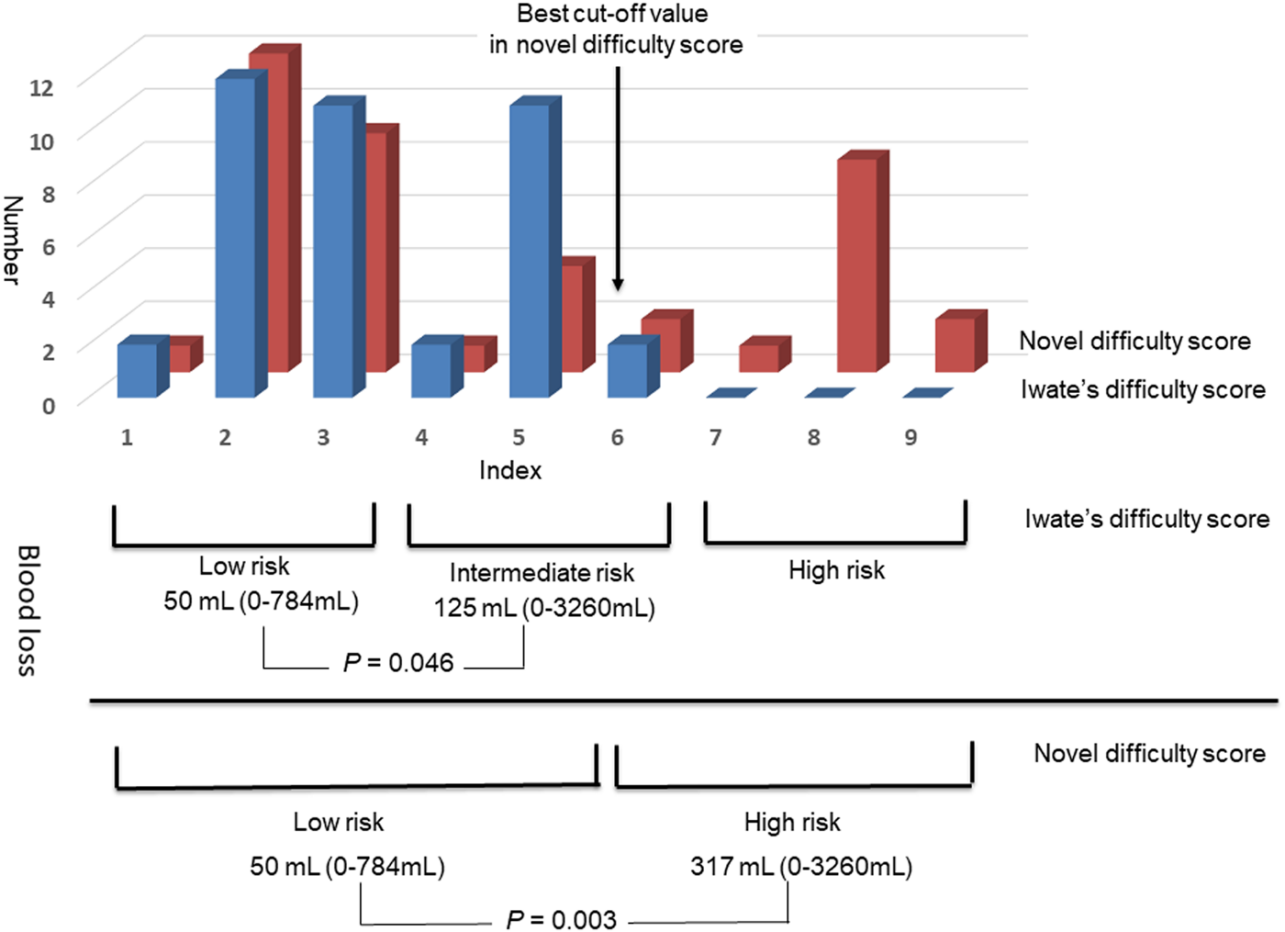
Distribution in accordance with the Iwate and novel difficulty scoring systems and the comparison of blood loss among risk groups classified in accordance with the Iwate and novel difficulty scoring systems.

We created a novel difficulty scoring system based on the Iwate difficulty scoring system to add the presence of tumours located adjacent and cranial to the previous surgical site as a risk assessment factor. Assuming that the effect of the risk factor (presence of tumours located adjacent and cranial or dorsal to the previous surgical site) on the difficulty of LRH was given a score of 3, the distributions of the difficulty index scores in accordance with the Iwate and our novel difficulty scoring systems are shown in Fig. 2. When cut-off scores of ≥4, 5, 6, and 7 were used to define the high-risk LRH group, this resulted in the distribution of 18 (43.9%), 17 (41.5%), 13 (32.5%), and 11 (26.8%) patients, respectively, into the high-risk LRH group, with respective *P* values of 0.034, 0.015, 0.003, and 0.009 for comparisons of intraoperative blood loss volume. Moreover, when the factor affecting the difficulty of LRH was given a score of 1, 2, or 4, *P* values of less than 0.003 were not obtained, regardless of where the cut-off point was set. Therefore, we defined the optimal cut-off value for the high-risk LRH group as 6 in the novel difficulty scoring system. However, the rate of conversion to laparotomy did not significantly change, regardless of where the cut-off point was set.

## Discussion

The present study showed that the presence of tumours located adjacent and cranial or dorsal to the previous surgical site, which was a factor related to massive blood loss in open repeat hepatectomy, was also useful in assessing the difficulty of LRH. Furthermore, our novel difficulty scoring system based on the Iwate difficulty scoring system was useful for predicting massive blood loss in LRH.

While most studies show that LRH is associated with less blood loss, fewer postoperative complications, and a shorter duration of postoperative hospitalisation compared with conventional open hepatectomy^6–10^, to our knowledge, the present study is the first to evaluate the use of these factors to predict the difficulty of LRH. Consistent with previous results^6–10^, our results showed that LRH resulted in less intraoperative blood loss and a shorter duration of postoperative hospitalisation compared with open repeat hepatectomy. In our study, we found that surgeons tended to avoid performing LRH in patients with tumours located in segments 1, 7 or 8 and patients with tumours ipsilateral to the previous surgical site, as the difficulty of LRH may be high in these patients. Therefore, there is an urgent need for a difficulty scoring system for LRH.

Ban *et al*.^11^ proposed the Iwate difficulty scoring system to assess the difficulty of LLR in 2014, and subsequent studies have validated this scoring system and confirmed its usefulness^12–15^. When the Iwate difficulty scoring system is used to classify patients into low-, intermediate-, and high-risk groups, the intraoperative blood loss volume and operation time reportedly significantly differ between the low- and high-risk groups and between the intermediate- and high-risk groups, but not between the low- and intermediate-risk groups^11^. However, the Iwate difficulty scoring system does not include factors related to repeat hepatectomy and tumours located in segment 1, as the presence of tumours in segment 1 was considered a contraindication for LLH when this procedure was first introduced.

Five years have passed since Ban *et al*.^11^ published the Iwate difficulty scoring system. In that time, the indications for LLR have been expanded, and LRH has been introduced. Therefore, it is now important to determine how much weight should be assigned using the Iwate difficulty scoring system to the factors affecting patients undergoing repeat hepatectomy or patients with tumours located in segment 1. In the current study, although no patients had a difficulty index of ≥7, which corresponds to the high-risk group in the Iwate difficulty scoring system, the intraoperative blood loss volume significantly differed between the low- and intermediate-risk groups, which was not seen in the original study by Ban *et al*.^11^ In the original study, the intermediate-risk group was defined as having a difficulty index of 4–6, while the high-risk group was defined as having a difficulty index of ≥7^11^. Comparing our results with those of Ban *et al*.^11^, the factor “repeat hepatectomy” may be equivalent to a score ≥3, as this factor resulted in a significant difference in intraoperative blood loss between our low- and intermediate-risk groups.

In the current study, we aimed to identify novel risk factors that predicted the difficulty of LRH, and we evaluated the recurrent tumour location using three methods. Our results showed that the presence of tumours located adjacent and cranial or dorsal to the previous surgical site, which was an independent risk factor for massive intraoperative blood loss in open repeat hepatectomy, was also a risk factor for blood loss in LRH, even though the incidence of this factor did not significantly differ between those who underwent LRH versus open repeat hepatectomy.

When comparing LRH and open repeat hepatectomy, we found that the selection of LRH or open hepatectomy depended on the presence of tumours located in segments 1, 7, or 8 and/or the presence of tumours ipsilateral to the previous surgical site, whereas the presence of tumours adjacent and cranial or dorsal to the previous surgical site was a more important factor when determining the difficulty of repeat hepatectomy. Therefore, if it is necessary to detach the surface adhesions resulting from previous liver resection, attention must be paid to the presence of tumours in segments other than S7 and/or S8, while LRH could be strongly considered for tumours located in segments 7 and/or 8 if adhesiolysis of the surface of the previous liver resection site is not necessary, as laparoscopy does not require the dissection of all adhesions, unlike in open surgery; adhesiolysis is performed only to access the operative field for hepatectomy in LRH.

When comparing the Iwate and novel difficulty scoring systems, the index for the low-risk group in the Iwate difficulty scoring system was almost the same in the novel difficulty scoring system, whereas the intermediate-risk group in the Iwate difficulty scoring system had a higher index in the novel difficulty scoring system. This suggests that there are many opportunities to detach surface adhesions at the previous liver resection site in cases with tumours located in segments 7 or 8. Almost all patients with a score of more than 6 in the novel difficulty scoring system had tumours located in segments 7 or 8 and tumours cranial or dorsal to the previous surgical site.

The present study had several limitations, including the retrospective design, small sample size, and single centre setting. Although the number of patients undergoing LRH in our study was larger than in previous reports^6–10^, further prospective multi-institutional studies are needed to validate our results. Another limitation is that we defined patients in the ≥75th percentile for blood loss volume as our high-risk group, and the results might differ depending on which factor and/or cut-off point is used to define high-risk patients among the several patient risk factors for LRH.

In conclusion, our results verified the usefulness of the Iwate difficulty scoring system to predict massive blood loss and identified the patient factors that predicted the difficulty of LRH. Our results suggest the usefulness of our novel difficulty scoring system for LRH based on the Iwate difficulty scoring system.

## Methods

### Patients and methods

During the time period from the introduction of LLR in our institution in April 2011 until December 2018, 194 patients underwent repeat hepatectomy. Among these patients, we evaluated data from 137 patients who underwent partial hepatectomy or left lateral sectionectomy without thoracotomy; the laparoscopic approach was avoided for patients with recurrence who required anatomical resection except in those requiring left lateral sectionectomy or thoracotomy because of invasion to the diaphragm. We retrospectively reviewed the database of our institution from inception until January 2019. As this was a retrospective study, the Institutional Review Board of Shizuoka Cancer Center waived the need for patient consent (number: J2019-59-2019-1-3).

All included patients underwent preoperative abdominal ultrasonography, computed tomography, gadolinium ethoxybenzyl diethylene-triamine-pentaacetic-acid-enhanced magnetic resonance imaging, and laboratory assessment of liver function. We assessed liver function using the Child-Pugh classification^16^ and liver damage criteria^17^ including the indocyanine green retention rate at 15 minutes. The indications for repeat hepatectomy were determined based on liver function and tumour location. Patients with early recurrence within 6 months were not candidates for repeat hepatectomy. Systemic chemotherapy was prescribed for patients with recurrent liver metastasis, while patients with recurrent HCC received non-surgical treatment such as transcatheter arterial chemoembolization, radiofrequency ablation, or sorafenib. The surgical procedure and extent of hepatectomy in each patient were determined during a weekly surgical conference.

We defined postoperative mortality as all in-hospital postoperative deaths, and classified complications into six grades in accordance with the modified Clavien-Dindo classification system^18^. When patients had undergone both laparoscopic and open previous hepatectomy, the approach to the previous hepatectomy was considered as open. We defined resection margins as R0 (tumour-free), R1 (microscopic tumour involvement), and R2 (macroscopic tumour involvement).

### Surgical procedure in LRH

The first trocar for laparoscopy was inserted via a small laparotomy to avoid injuring the intra-abdominal organs affected by adhesions from the previous hepatectomy. After establishing pneumoperitoneum, three or four additional trocars were inserted. In most cases, it was possible to proceed with the operation, but sometimes one or two additional trocars were needed for adhesiolysis. Intraoperative ultrasonography was routinely performed to assess the tumours, understand the intrahepatic structures, and determine the transection line. The Pringle manoeuvre was routinely performed for as long as possible, even in repeat hepatectomy. However, the Pringle manoeuvre was abandoned when severe adhesions were present around the hepatoduodenal ligament. Hepatectomy was performed using an ultrasonic coagulation and incision system (HARMONIC^®^ HD 1000i Laparoscopic Shears; Ethicon, Inc., Cincinnati, OH, USA) and a ball-type IO-advance electrode (VIO 300D; ERBE Elektromedizin, Tubingen, Germany).

### Surgical procedure in open repeat hepatectomy

Open hepatectomy was routinely performed through a midline or a midline and subcostal incision, with thoracotomy added if needed. Hepatectomy was performed using an ultrasonic surgical aspirator (CUSA Excel; Integra LifeSciences, Plainsboro, NJ, USA) and an ultrasonic coagulation and incision apparatus chosen by the attending surgeon.

### Validation of the usefulness of the difficulty scoring system proposed by Ban *et al*.11 in LRH

Patient clinical factors were compared between the low-, intermediate-, and high-risk groups classified in accordance with the difficulty scoring system of Ban *et al*.^11^

### Recurrent tumour location relative to the previous surgical site

**Figure 3:**
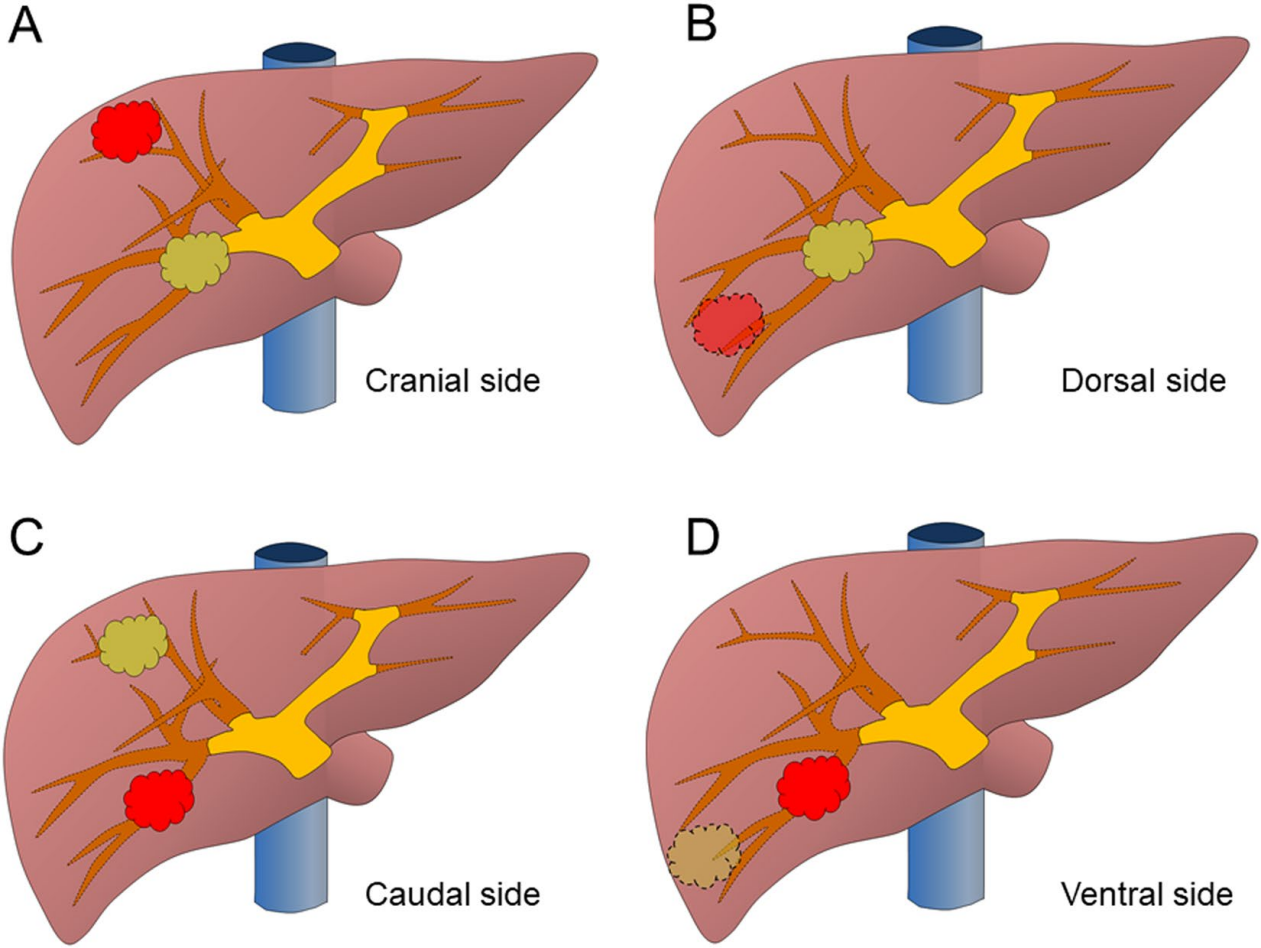
The positional relationship between the recurrent tumor and the previous tumor. The recurrent tumor is shown in red. (**A**) A recurrent tumor located adjacent and cranial to the previous surgical site. (**B**) A recurrent tumor located adjacent and dorsal to the previous surgical site. (**C**) A recurrent tumor located adjacent and caudal to the previous surgical site. (**D**) A recurrent tumor located adjacent and ventral to of the previous surgical site.

The recurrent tumour location was evaluated using three methods. First, we evaluated whether the recurrent tumour was located in segments 1, 7, or 8, as it is difficult to perform LLR for these segments. Second, we evaluated whether the location of the recurrent tumour was ipsilateral to the previous surgical site, as adhesion dissection can be omitted if the recurrent tumour is located contralateral to the previous surgical site. Finally, we evaluated whether the recurrent tumour was located adjacent to the original site cranially (Fig. 3A) or dorsally (Fig. 3B), as adhesion dissection is also omitted if the recurrent tumour is located adjacent and caudal (Fig. 3C) or ventral (Fig. 3D) to the previous surgical site.

### Definitions of the factors affecting massive blood loss in open repeat hepatectomy

In accordance with the difficulty scoring system for LLR^11^, blood loss volume and operation time were increased in the high-risk group. We defined the risk factors for massive intraoperative blood loss as those present in patients in the ≥75th percentile for blood loss volume in open repeat hepatectomy. We then examined whether these extracted risk factors were useful for risk assessment in LRH when combined with the Iwate difficulty scoring system^11^.

### Statistical analyses

Continuous variables are presented as the median and range and were compared using the Mann-Whitney U test. Categorical variables were compared using the chi-squared test or Fisher’s exact test, as appropriate. The cut-off points for the laboratory data were defined as the upper limits of normal applied at our institution, while the cut-off points for age and tumour diameter were defined as the respective median values. Cumulative relapse-free and overall survival curves were analysed using the Kaplan-Meier method and compared using the log-rank test. Binomial logistic regression was used for the uni- and multivariate analyses, and all factors found to be significant predictors of massive blood loss in LRH (*P* < 0.05) in the univariate analysis were entered into the multivariate analysis. All statistical analyses were performed using the SPSS 24.0 software package (IBM Corp., Armonk, NY, USA), and two-tailed *P* values of < 0.05 were considered to indicate statistical significance.

### Ethical approval

This study confirmed to the ethical guidelines of the Declaration of Helsinki (2013 revision) and was retrospective in nature, and we obtained approval from the Institutional Review Board of Shizuoka Cancer Center (number: J2019-59-2019-1-3).
